# Effects of Citicoline, Homotaurine, and Vitamin E on Contrast Sensitivity and Visual-Related Quality of Life in Patients with Primary Open-Angle Glaucoma: A Preliminary Study

**DOI:** 10.3390/molecules25235614

**Published:** 2020-11-29

**Authors:** Pier Franco Marino, Gemma Caterina Maria Rossi, Giuseppe Campagna, Decio Capobianco, Ciro Costagliola, on behalf of QUALICOS Study Group

**Affiliations:** 1 Department of Medicine and Health Science “V. Tiberio”, the University of Molise, 86100 Campobasso, Italy; alpapini@tiscali.it; 2 IRCCS San Matteo Polyclinic Foundation, 27100 Pavia, Italy; gemma.rossi.md@gmail.com; 3 Department of Medical-Surgical Sciences and Translational Medicine, University of Rome “La Sapienza”, 00185 Rome, Italy; gius.campagna@gmail.com; 4 Ophthalmology Unit, Perimetry and Glaucoma Clinic, ASL NA1 e CE, 80035 Naples, Italy; deciocap@alice.it

**Keywords:** primary open-angle glaucoma, citicoline, homotaurine, vitamin E, contrast sensitivity, quality of life, SPARCS, neuroprotection, glaucoma medical treatment

## Abstract

The aim of the present study was to evaluate the effects of supplementation with a fixed combination of citicoline 500 mg, homotaurine 50 mg, and vitamin E 12 mg (CIT/HOMO/VITE) on contrast sensitivity and visual-related quality of life in patients with primary open-angle glaucoma (POAG) in mild stage. This was a multicenter, observational, cross-over, short-term, pilot study on POAG patients with stable controlled intraocular pressure (IOP). Patients were randomly assigned to Group 1 (current topical therapy for 4 months and then current topical therapy plus CIT/HOMO/VITE for 4 months) or Group 2 (CIT/HOMO/VITE in addition to current topical therapy for 4 months and then topical therapy alone for 4 months). Best-corrected visual acuity, IOP, visual field, and the Spaeth/Richman contrast sensitivity (SPARCS) test score were recorded at baseline and after 4 and 8 months. The Glaucoma Quality of Life-15 (GQL-15) questionnaire was administered at each check time. Forty-four patients were assigned to Group 1 and 65 to Group 2. Over the follow-up period, there were no significant changes in IOP or visual field findings, whereas SPARCS and GQL-15 findings significantly varied from baseline, both being improved in subjects treated with CIT/HOMO/VITE fixed combination. These results demonstrate that a daily intake of a fixed combination of citicoline, homotaurine, and vitamin E in addition to the topical medical treatment significantly increased the total score of the contrast sensitivity test and the quality of life in patients with POAG.

## 1. Introduction

The term glaucoma, far from representing a single and well-defined disease, encompasses a group of ocular conditions characterized by progressive optic nerve damage associated with a gradual visual field loss [1]. Primary open-angle glaucoma (POAG), the most common form, remains silent for years until damage to the optic nerve head and changes to the peripheral visual field occur [2]. Consequently, glaucoma is still one of the leading causes of irreversible blindness worldwide [3]. Although intraocular pressure (IOP) is the only proven treatable risk factor, lowering of IOP does not seem to halt progression in all cases; in some glaucomatous patients, retinal ganglion cell (RGC) loss may continue regardless of IOP reduction [4].

Due to the lack of specific symptoms, glaucoma is named the “silent thief of sight,” given that clinical signs occur only when the disease is quite advanced and can adversely affect the daily vision-related activities and the quality of life of patients [5]. The European Glaucoma Society has recognized that the goal of glaucoma care is the preservation of visual function and quality of life [6]. Visual function is not intended only as visual acuity but also as visual quality, i.e., even when visual acuity is within the normal range, patients with glaucoma may have an abnormal visual function that can affect their daily activities, mainly walking and driving [7], depending on the visual field defects (position and severity) and contrast sensitivity (CS) [8]. In particular, CS detects glaucomatous changes even before the occurrence of functional (visual field) and structural (optical coherence tomography, OCT) modifications [9], which in turn show a fair relationship with CS [10]. Besides, it has been demonstrated via CS tests that patients with mild to moderate glaucoma have poorer visual search ability, such as reading, driving, recognizing people, compared to age-matched nonglaucomatous subjects [11]. Despite the obvious importance of IOP in glaucoma development and progression, many patients with elevated IOP develop structural and functional characteristics of glaucoma at a very slow rate, whereas symptoms in patients whose IOP is well controlled worsen rapidly. This evidence strongly indicates that lowering IOP does not prevent progression in all patients; therefore, risk factors other than IOP are involved in the disease progression and form the rational basis for the current development of neuroprotection as a therapeutic strategy for glaucoma [12].

Several molecules have neuroprotective properties, and among them, citicoline (CIT) appears to be of interest and has been tested in different studies in ophthalmology and is supported by evidence from in vitro, in vivo, clinical, and randomized controlled trials [13]. Exogenous administration of CIT provides both choline and cytidine, which act as substrates for the synthesis of phosphatidylcholine, a primary neuronal membrane component, playing a role in the mechanisms of membrane repair and regeneration. Furthermore, CIT may alleviate free fatty acid-induced toxicity, which accompanies ischemic insult [14], and has been tested and used for years in patients with neurodegenerative diseases, such as glaucoma. In several clinical studies, its oral (500 mg) or intramuscular administration has been associated with an improvement in the functions of the retinal and visual pathways [15,16,17,18].

Homotaurine (3-amino-1-propane sulphonic acid, tramiprosate) (HOMO) is a compound of natural amino sulfonate with neuromodulatory and neuroprotective effects. HOMO, in in vitro and in vivo models, has been shown to possess the ability to interfere with the course of amyloid-related diseases by binding to the soluble amyloid protein. In POAG, it may act by preventing the aggregation and formation of amyloid plaques, also found at the level of RGCs, responsible for cell death [19]. Due to its affinity to GABA A receptors, it modulates cortical inhibitory activity by reducing the response of neurons to excitatory stimuli of glutamate [20,21], the pathogenic mechanism through which the RGCs and other nerve cells die as a consequence of an excess of extracellular glutamate [22].

Vitamin E (VITE) is a member of the family that contains lipid-soluble tocopherols and tocotriols and essential micronutrients with strong antioxidant activities. Among the family of VITE, α-tocopherol is the main constituent found in mammalian tissues and has the highest biological activity. In animal models, α-tocopherol has been shown to reduce the degeneration of hippocampal cells following cerebral ischemia and improve Alzheimer’s disease [23]. Rats with surgically induced IOP elevation on a VITE-deficient diet, experienced significantly more RGC death compared to that in rats under a normal diet. This effect was due to the increased level of lipid peroxidation in VITE-deficient rats [24]. In humans, a daily intake of VITE in patients affected by glaucoma was effective in reducing the progression of the disease [25]. The vital role that VITE plays in all tissues, including the eye, is its antioxidant effect [26,27].

Recently, a fixed combination of the abovementioned molecules became available: our preliminary study aimed to evaluate the effects of a fixed combination of CIT 500 mg, HOMO 50 mg, and VITE 12 mg on CS and vision-related quality of life in glaucomatous patients already on medical topical treatment.

## 2. Materials and Methods 

This multicenter, observational, cross-over, short-term, pilot study was conducted according to the recommendations of the Helsinki declaration (revision 2000, Edinburgh) and the Italian legislation on good clinical practices (DM 15 July 1997 and amendments). Approval was also obtained from the Technical Scientific Committee of the Department of Medicine and Health Science “V. Tiberio,” the University of Molise (prot. 14/2019).

All patients underwent a complete eye examination, including IOP measurement, gonioscopy, ophthalmoscopy, central corneal thickness measurement, standard automated white-on-white perimetry (SAP), the Spaeth/Richman contrast sensitivity (SPARCS) test, and completed the Glaucoma Quality of Life-15 (GQL-15) questionnaire (Supplementary Materials). IOP measurement (Goldmann applanation tonometry, GAT) was always performed in the morning (between 8:00 and 10:00 a.m.); SAP was performed with a Humphrey Field Analyzer (HFAII, Carl Zeiss Meditec, Dublin, CA, USA), with a size III stimulus, Swedish interactive threshold algorithm (SITA) standard, and 30-2 pattern. Only exams with good reliability indices (with less than 33% fixation losses or false-negative errors or less than 15% false-positive errors) were included in the study for statistical evaluation.

The SPARCS test is a CS test that can be performed on a computer with access to the Internet; the test was chosen precisely for this characteristic that made it available to all the centers participating in the study. The SPARCS test evaluates patients’ central vision and peripheral vision. It was designed to provide information on the aspect of patients’ vision that is highly related to daily life. The test was developed and made available to detect CS in patients with glaucoma [28,29]. The test was performed on a standard computer with a monitor set to 1024 × 768 resolution, 256 gray levels, and a size of at least 22 cm width and 26.5 cm height. Patients are seated 50 cm from the computer monitor. At this testing distance, the test occupies 30° of vision horizontally and 23.5° of vision vertically. The central test area subtends 5° horizontally and 3.5° vertically. Patients are instructed to fixate on the central area and identify which of the areas appears different. When patients are ready, they click on the central area. Vertical square wave gratings with a spatial frequency of 0.4 cycles per degree appear for 0.3 s in one of the five tested areas. The range of contrast tested is from 100% to 0.45% (log contrast sensitivity 0.00 to 2.35) and decreases by approximately 0.15 log units between levels. The contrast value is calculated by Weber contrast. The central area and four peripheral areas each receive separate scores. Each log-based score is then scaled out of 20 by dividing by 2.35 and multiplying by 20. A total SPARCS score is summated from each of the five areas, making 100 the perfect summed score from all five areas [30]. The test, for both eyes, typically takes 10 min. SPARCS has high test–retest reliability in identifying individuals with glaucoma; the score in normal subjects is between 52.9 and 87.2 [31]. However, to minimize the learning effect, each patient performed three practice tests at t0, before randomization. In the present study, for simplicity and simplification, the researchers collected and analyzed only the average data provided at the end of the test, and the CS was measured binocularly.

**Figure 1:**
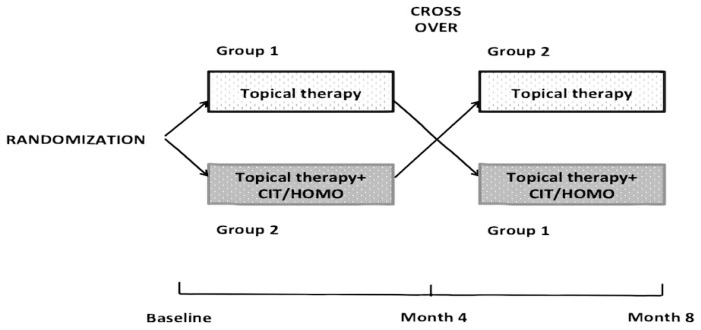
Study design. After randomization, patients were assigned to Group 1 (current topical Intraocular Pressure (IOP) lowering therapy) or Group 2 (current topical therapy plus one tablet per day of CIT/HOMO-citicoline and homotaurine fixed). Four months later, patients of Group 1 started with CIT/HOMO tablet in addition to current topical IOP lowering therapy, whereas patients of Group 2 continued with the current topical IOP lowering therapy alone.

The GQL-15 is a questionnaire based on 15 rating-scored questions that evaluate the degree of functional disability caused by glaucoma, i.e., six questions are related to actions that require functional peripheral vision, six to dark adaptation and glare, two to central and near vision, and one to outdoor mobility. The score ranges from 0 to 75, higher scores indicating poorer quality of life [32]. IOP lowering topical therapy included 0.5 mg of timolol (31 patients, 28.97%), prostaglandin analogues and prostamides (36 patients, 33.64%), carbonic anhydrase inhibitors (CAI, 3 patients, 2.80%), fixed combination timolol and prostaglandin (21 patients, 19.63%), fixed combination timolol and CAI (3 patients, 2.80%), CAI and prostaglandin (3 patients, 2.80%), timolol, and CAI and prostaglandin (10 patients, 9.35%). The eligibility criteria were (a) age 18 years or older, (b) POAG diagnosis, (c) stable controlled IOP (<18 mmHg, morning value) with beta-blocker or prostaglandin analogues in the last 6 months, and (d) SAP mean deviation (MD) reduction ranging between 1 and 1.5 db/year during the previous 2 years. Exclusion criteria were (a) inability to perform SAP, (b) best-corrected visual acuity (BCVA) worse than 0.5 decimals, (c) significant ocular media opacities, (d) history of previous eye surgery, and (e) concomitance with systemic diseases that might lead to visual acuity damage or affect SAP execution. All eligible patients gave their written informed consent after being provided with a detailed description of the objectives of the study and the procedure to be used. Investigations were conducted following Good Clinical Practice. Included patients were randomly divided into two groups: Group 1 continued current topical IOP lowering therapy alone for the first 4 months, after which they received one tablet per day of the fixed combination CIT/HOMO/VITE in addition to the current topical therapy for the next 4 months. Group 2 received one tablet per day of the fixed combination CIT/HOMO/VITE in addition to the current topical IOP lowering therapy for 4 months and then continued with the current topical therapy alone for the next 4 months. The study design is summarized in Figure 1.

All patients were examined at baseline (t0), after 4 (t4), and 8 (t8) months. At each check, visual acuity, IOP, SAP, and SPARCS test scores were recorded, and the GQL-15 questionnaire was administered.

During the follow-up period (8 months), no IOP spikes occurred, with a mean IOP increase in no more than 3 mmHg at every check. The CIT/HOMO/VITE fixed combination Neuprozin^®^ (citicoline 500 mg, homotaurine 50 mg, and vitamin E 12 mg) was provided by FB-Vision (Via Piceno Aprutina, 47 63,100 Ascoli Piceno, Italy). For statistical analysis, the eye with the best visual acuity was chosen for each patient, the right eye was examined when the visual acuity was the same in both eyes.

### Statistical Analysis

Statistical analyses were performed using SAS v. 9.4, JMP v. 15 (SAS, Institute Inc., Cary, NC, USA). Categorical variables were expressed as absolute frequencies and percentages. Continuous variables were presented as median (minimum to maximum). The normality was tested by the Shapiro–Wilk test. The Wilcoxon test was used in intragroup analyses to test the differences between the single temporal points (baseline (t0) vs. 4 months (t4) and 4 months (t4) vs. 8 months (t8)) of quality of life (GQL-15) score and CS (SPARCS) score. Spearman correlation (data non-normal distribution) was used to assess the correlations between the continuous variables. For continuous variables, median values among Group 1 (Current topical IOP lowering therapy) and Group 2 (Current topical IOP lowering therapy + one tablet per day of the fixed combination CIT/HOMO/VITE) were compared by the Mann–Whitney *U* test when the data were not normally distributed. The frequencies were compared using the χ^2^ test or Fisher’s exact test when appropriate. Cross-over analysis was performed by a mixed model (Grizzle’s model). This model evaluated the differences between Group 1 and Group 2 and the effect of carryover (absent when *p* > 0.05). Homoscedasticity was tested through checking of the studentized residuals. A probability level of *p* ≤ 0.05 was considered statistically detectable.

## 3. Results

One hundred and nine patients with POAG were recruited for the study. The baseline characteristics of the enrolled patients are reported in Table 1. Forty-four patients (40.36%) were randomly assigned to Group 1 and 65 (59.63%) to Group 2. Five patients were lost during the study (3 in the Group 1 and 2 in the Group 2), but the baseline characteristics being the same between the two groups does not generate bias in the results. At baseline, clinical characteristics were similar between the two groups, with no statistical differences (Table 2). At this time check, a negative significant correlation was observed between the quality of life score and visual field defect (ρ = −0.26; *p* = 0.0003), whereas no correlation was detected between the quality of life and years from POAG diagnosis (ρ = −0.04; *p* = 0.61). No correlation was also found between quality of life and CS (ρ = −0.07; *p* = 0.35) and between CS and visual field (ρ = −0.03; *p* = 0.68). Neither significant changes in IOP values nor significant effects on visual field parameters were derived from the cross-over analysis.

**Figure 2:**
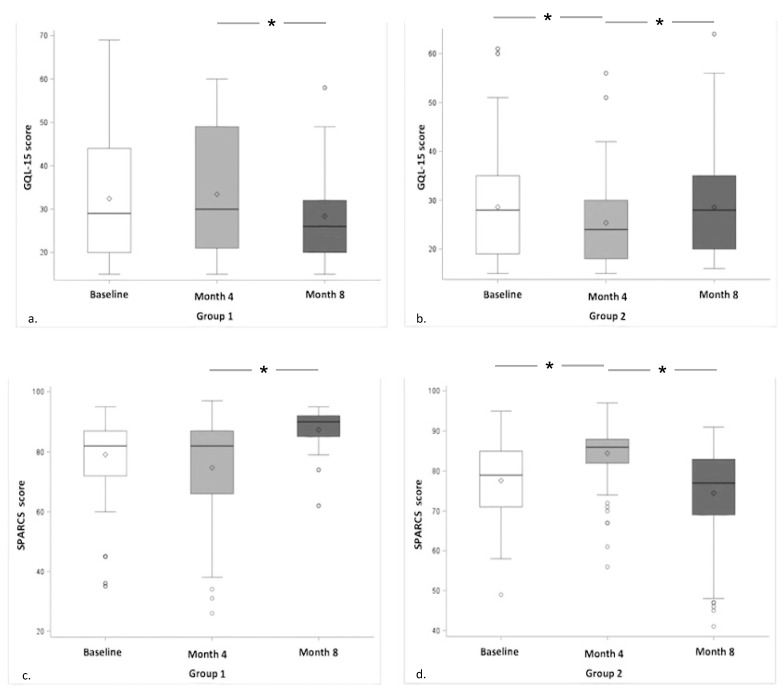
Quality of life (GQL-15 score) and contrast sensitivity (SPARCS score) changes over the follow-up period (baseline, month 4 and month 8). (**a**,**b**) Quality of life examined with GQL-15 questionnaire in Group 1 and Group 2 respectively. (**c**,**d**) Contrast sensitivity examined with SPARCS in Group 1 and Group 2 respectively. * *p* < 0.0001 for all.

Contrarily, significant changes were recorded for both quality of life and CS; these data are summarized in Figure 2. The quality of life, examined with the GQL-15 questionnaire, improved in both groups during the 4 months of daily intake of one tablet per day of CIT/HOMO/VITE. In effect, the score changed from 30 (21 to 49) to 26 (20 to 32) in Group 1 (*p* < 0.0001) and from 28 (19 to 35) to 24 (18 to 30) in Group 2 (*p* < 0.0001). Similarly, CS, examined by the SPARCS total score, improved in both groups during the 4 months of daily intake of CIT/HOMO/VITE increasing from 82 (66 to 87) to 90 (85 to 92) in Group 1 (*p* < 0.0001) and from 79 (71 to 85) to 85.5 (82 to 88) in Group 2 (*p* < 0.0001) (Figure 2).

Table 3 reports the cross-over analysis; a significant difference was observed between the treatments (Group 1 = topical IOP lowering therapy only compared to Group 2 = topical therapy plus one tablet *per* day of CIT/HOMO/VITE) in relation to GQL-15 (*p* < 0.0001) and SPARCS total score (*p* = 0.0004). The GQL-15 total score was lower (therefore, a better quality of life) in subjects treated with IOP lowering therapy plus one tablet per day of the CIT/HOMO/VITE fixed combination for 4 months. Additionally, the SPARCS score improved over the same period. The nonsignificance of the sequence (Group 1/Group 2 vs. Group 2/Group 1) for both the GQL-15 score and SPARCS score indicates no carryover effect (Table 3).

All patients, during the period of CIT/HOMO/VITE intake, were asked about the occurrence of side effects. Table 4 shows that the side effects reported by patients during the study were not related to the CIT/HOMO/VITE intake.

## 4. Discussion

These preliminary findings demonstrate that the daily intake of a fixed combination of CIT 500 mg, HOMO 50 mg, and VITE 12 mg for 4 months, in addition to the current topical therapy for the reduction in IOP, improves both the CS and the quality of life in patients with POAG. No effects on visual field parameters were observed.

Glaucoma is a chronic disease with negative consequences on the quality of life of patients [33]. It influences the quantity and quality of vision, by limiting the daily activities of the affected patients, according to the severity of the disease. Several previous studies have shown that patients with glaucoma and glaucomatous visual field loss have lower quality of life questionnaire scores compared to those obtained by healthy subjects [34]. The evidence was so high that the European Glaucoma Society recognized that the goal of treatment is the preservation of visual function and quality of life [6]. Although it is clear that quality of life is the goal of glaucoma treatment, the methods of improving this are not fully understood. Some studies have suggested that some therapeutic choices (pharmacological, laser, or surgical) [35,36] may improve this parameter. Contrarily, other studies have shown that great attention must be paid to concomitant disorders related to the use of treatments for IOP control, mainly the onset of a dry eye syndrome that can contribute to worsening the quality of life in these patients [37,38].

The challenge is, therefore, to identify molecules that can improve patients’ quality of life. Our study has shown that the tested molecules can improve the quality of life as examined with the GQL-15 questionnaire, while equally ameliorating vision quality, as ascertained by CS. CS is not routinely assessed in glaucomatous patients, despite its important role in daily functioning and activities [39]. Recently, Montana et al. found that the main factors influencing daily activities are not only the severity and location of visual field defects but also CS and the performance in attention tasks [7]. In the early stages of glaucomatous disease, CS, as well as visual acuity, is not affected, since it reflects central visual function, while glaucoma initially affects the peripheral visual field. In addition, glaucoma is a symmetric but asynchronous disease that can affect both eyes differently. In binocular vision, patients with glaucoma can compensate for the deficiency of one eye with the other, justifying the great adaptability of glaucomatous patients while performing daily activities. A few studies exist on the effect of CIT on CS. Notwithstanding, the precise mechanism of action is unclear. This is probably due to both its dopaminergic action and a direct retinal action that improves latency in pattern electroretinogram examination [15,40]. As a matter of fact, it was observed that in rabbits treated with CIT, the concentration of dopamine in the retina increased [41].

A PubMed search did not offer data on the effects of HOMO on CS, although a cytoprotective action, performed through its effects on oxidative damage, was well ascertained [42]. A recent in vitro study has demonstrated the synergistic cytoprotective effects of co-treatment with CIT and HOMO on cultured retinal cells [43]. Based on these data, the present study examined the effects of this fixed association in vivo. To the best of our knowledge, no previous studies have evaluated the effect of HOMO 50 mg plus CIT 500 mg, either on CS or on the quality of life.

Finally, a significant increase in oxidative stress may play a role in the pathogenesis of POAG. The determination of oxidative stress in the aqueous humor of these patients has revealed a significant decrease in the levels of vitamins C and E, suggesting that treatment with antioxidants may represent a relevant preventive and therapeutic approach [44].

The strengths of this study are (1) the randomized cross-over design; (2) the evaluation of the quality of life; (3) the evaluation of the quality of vision, which are important outcomes in glaucomatous patients; and (4) the absence of side effects. Besides, this multicenter collaborative research method together with the utilization of a simple protocol in real life context reduces the possibility of bias related to both a single-center design and the performance of complex tests. Over the follow-up period there were not significant changes in IOP responsible for the occurrence of systemic symptoms.

However, we recognize and hope that our results are to be confirmed by clinical trials with more robust and strong designs; in fact, the limitations of the present study include the lack of a placebo group, a small sample size, and a short-term follow-up. Moreover, we cannot determine, with the present results, which component is responsible of the improvement seen in CS and GQL; further studies on the effects of the individual components will be needed. 

## 5. Conclusions

In conclusion, data from this preliminary study strongly suggest that concomitant therapy with CIT 500 mg, HOMO 50 mg, and VITE 12 mg can have a positive impact on the quality of vision and quality of life of patients with mild stage glaucoma. This association could represent a potential new option with an ability to improve the health status and daily life performances in these patients.

## Figures and Tables

**Table 1:** Baseline demographic and clinical characteristics.

Parameter	*n* (%)
Sex	
Female	57 (52.29)
Male	52 (47.61)
Years since diagnosis of glaucoma * (years)	7 (1 to 19)
Current topical therapy	
Monotherapy	72 (66.06)
Multitherapy	37 (33.94)
Number of Intraocular Pressure (IOP) lowering eye-drop bottles per day	
One bottle	88 (80.73)
Two bottles	20 (18.35)
Three bottles	1 (0.92)
Type of current therapy	
Timolol	31 (28.44)
Carbonic Anhydrase Inhibitors (CAI)	3 (2.75)
Prostaglandin (PG)	36 (33.02)
Timolol + PG	21 (19.26)
Timolol + CAI	5 (4,59)
CAI + PG	3 (2.75)
Timolol + CAI + PG	10 (9.17)
Contrast sensitivity (SPARCS) * (score)	80 (35 to 95)
Quality of life (GQL-15) * (score)	28 (15 to 69)
Best Corrected Visual Acuity (BCVA) * (decimals)	1.0 (0.5 to 1.0)
Intraocular pressure (IOP) * (mmHg)	16 (10 to 25)
Visual field * (mean deviation)	−1.72 (−19.00 to 3.18)

* Data presented by median (minimum to maximum).

**Table 2:** Differences between groups at randomization and the baseline demographic and clinical characteristics.

Parameter	Group 1	Group 2	*p*
Age (years)	66.5 (45 to 79)	68.0 (48 to 85)	0.57
Sex			0.051
Female	18 (40.91)	39 (60.00)	
Male	26 (59.09)	26 (40.00)	
Years since diagnosis of glaucoma (years)	5.5 (1 to 19)	7.0 (1 to 19)	0.41
Current topical therapy			0.74
Monotherapy	28 (63.64)	43 (66.15)	
Multitherapy	16 (36.36)	22 (33.85)	
Number of IOP lowering eye-drop bottles per day			1.00
One bottle	37 (84.09)	52 (80.00)	
Two bottles	7 (15.90)	12 (18.46)	
Three bottles	0 (0.00)	1 (1.54)	
Type of current therapy			0.99
Timolol	12 (27.27)	19 (29.23)	
Carbonic Anhydrase Inhibitors (CAI)	1 (2.27)	2 (3.08)	
Prostaglandin (PG)	15 (34.09)	22 (33.84)	
Timolol + CAI	2 (4.55)	3 (4.61)	
Timolol + PG	9 (20.45)	11 (16.92)	
CAI + PG	1 (2.27)	2 (3.08)	
Timolol + CAI + PG	4 (9.09)	6 (9.22)	
Contrast sensitivity (SPARCS) (score)	82 (35 to 95)	79 (49 to 95)	0.06
Quality of life (GQL-15) (score)	29 (15 to 69)	28 (15 to 61)	0.17
Best Corrected Visual Acuity (BCVA) (decimals)	1.0 (0.6 to 1.0)	1.0 (0.5 to 1.0)	0.21
Intraocular pressure (IOP) (mmHg)	16 (10 to 25)	16 (11 to 22)	0.41
Visual field (mean deviation)	−1.53 (−13.96 to 3.18)	−1.84 (−19.00 to 1.94)	0.15

Group 1: Current topical intraocular pressure (IOP) lowering therapy. Group 2: Current topical intraocular pressure (IOP) lowering therapy + one tablet per day of the fixed combination CIT/HOMO/VITE.

**Table 3:** Estimations of fixed effects based on Grizzle’s method.

Parameter	Beta ± SE	Beta ± SE	*p*
Quality of life (GQL-15) (score)			
Group 1 vs. Group 2	30.75 ± 0.94	28.62 ± 0.94	<0.0001
Group 1/Group 2 vs. Group 2/Group 1	30.64 ± 1.46	28.72 ± 1.08	0.29
Contrast sensitivity (SPARCS) (score)			
Group 1 vs. Group 2	79.14 ± 0.78	83.05 ± 0.79	0.0004
Group 1/Group 2 vs. Group 2/Group 1	80.58 ± 0.68	81.61 ± 0.93	0.19
Visual field (mean deviation)			
Group 1 vs. Group 2	−2.59 ± 0.42	−2.72 ± 0.42	0.55
Group 1/Group 2 vs. Group 2/Group 1	−2.18 ± 0.66	−3.13 ± 0.48	0.24

The bold is for the statistically significant values.

**Table 4:** Distributions of side effects.

Side Effects	*n* * (%)	*n* ** (%)
Anxiety	3 (15.79)	3 (2.75)
Headache	2 (10.53)	2 (1.83)
Sleep disorders	6 (31.58)	6 (5.50)
Irritability	4 (21.05)	4 (3.67)
Slight weight gain	1 (5.26)	1 (0.92)
Nausea	2 (10.53)	2 (1.83)
Tachycardia	1 (5.26)	1 (0.92)

* percentages calculated by *n* = 19, ** percentages calculated by *n* = 109.

## Supplementary Materials

The following are available online, Glaucoma Quality of Life-15 (GQL-15) questionnaire.

## References

[1] Costagliola C., Dell’Omo R., Romano M.R., Rinaldi M., Zeppa L., Parmeggiani F. (2009). Pharmacotherapy of intraocular pressure: Part I. Parasympathomimetic, sympathomimetic and sympatholytics. Expert Opin. Pharmacother..

[2] Grzybowski A., Och M., Kanclerz P., Leffler C.T., De Moraes C.G. (2020). Primary Open Angle Glaucoma and Vascular Risk Factors: A Review of Population Based Studies from 1990 to 2019. J. Clin. Med..

[3] Quigley H. (1996). Number of people with glaucoma worldwide. Am. J. Ophthalmol..

[4] Weinreb R.N., Khaw P.T. (2004). Primary open-angle glaucoma. Lancet.

[5] Daga F.B., Gracitelli C.P.B., Diniz-Filho A., Medeiros F.A. (2018). Is vision-related quality of life impaired in patients with preperimetric glaucoma?. Br. J. Ophthalmol..

[6] (2014). Terminology and Guidelines for Glaucoma.

[7] Montana C.L., Bhorade A.M. (2018). Glaucoma and quality of life. Curr. Opin. Ophthalmol..

[8] Onal S., Yenice O., Cakir S., Temel A. (2007). FACT contrast sensitivity as a diagnostic tool in glaucoma. Int. Ophthalmol..

[9] Shoshani Y.Z., Harris A., Rusia D., Spaeth G.L., Siesky B., Pollack A., Wirostko B. (2011). Contrast sensitivity, ocular blood flow and their potential role in assessing ischaemic retinal disease. Acta Ophthalmol..

[10] Fatehi N., Nowroozizadeh S., Henry S., Coleman A.L., Caprioli J., Nouri-Mahdavi K. (2017). Association of Structural and Functional Measures With Contrast Sensitivity in Glaucoma. Am. J. Ophthalmol..

[11] Lee S.S.-Y., Wood J.M., Black A.A. (2020). Impact of glaucoma on executive function and visual search. Ophthalmic Physiol. Opt..

[12] Nucci C., Martucci A., Giannini C., Morrone L.A., Bagetta G., Mancino R. (2018). Neuroprotective agents in the management of glaucoma. Eye.

[13] Gandolfi S., Marchini G., Caporossi A., Scuderi G., Tomasso L., Brunoro A. (2020). Cytidine 5′-Diphosphocholine (Citicoline): Evidence for a Neuroprotective Role in Glaucoma. Nutrients.

[14] D’Orlando K.J., Sandage B.W. (1995). Citicoline (CDP-Choline): Mechanisms of action and effects in ischemic brain injury. Neurol. Res..

[15] Porciatti V., Schiavi C., Benedetti P., Baldi A., Campos E.C. (1998). Cytidine-5’-diphosphocholine improves visual acuity, contrast sensitivity and visually-evoked potentials of amblyopic subjects. Curr. Eye Res..

[16] Roberti G., Tanga L., Michelessi M., Quaranta L., Parisi V., Manni G., Oddone F. (2015). Cytidine 5′-Diphosphocholine (Citicoline) in Glaucoma: Rationale of Its Use, Current Evidence and Future Perspectives. Int. J. Mol. Sci..

[17] (2018). Frontiers in Aging Neuroscience. Front. Aging Neurosci..

[18] Lanza M., Carnevale U.A.G., Mele L., Sconocchia M.B., Bartollino S., Costagliola C. (2019). Morphological and Functional Evaluation of Oral Citicoline Therapy in Chronic Open-Angle Glaucoma Patients: A Pilot Study With a 2-Year Follow-Up. Front. Pharmacol..

[19] Caltagirone C., Ferrannini L., Marchionni N., Nappi G., Scapagnini G., Trabucchi M. (2012). The potential protective effect of tramiprosate (homotaurine) against Alzheimer’s disease: A review. Aging Clin. Exp. Res..

[20] Bossù P., Salani F., Ciaramella A., Sacchinelli E., Mosca A., Banaj N., Assogna F., Orfei M.D., Caltagirone C., Gianni W. (2018). Anti-inflammatory Effects of Homotaurine in Patients With Amnestic Mild Cognitive Impairment. Front. Aging Neurosci..

[21] Russo R., Adornetto A., Cavaliere F., Varano G.P., Rusciano D., Morrone L.A., Corasaniti M.T., Bagetta G., Nucci C. (2015). Intravitreal injection of forskolin, homotaurine, and L-carnosine affords neuroprotection to retinal ganglion cells following retinal ischemic injury. Mol. Vis..

[22] Rolle T., Dallorto L., Rossatto S., Curto D., Nuzzi R. (2020). Assessing the Performance of Daily Intake of a Homotaurine, Carnosine, Forskolin, Vitamin B2, Vitamin B6, and Magnesium Based Food Supplement for the Maintenance of Visual Function in Patients with Primary Open Angle Glaucoma. J. Ophthalmol..

[23] Acar S.E., Sarıcaoğlu M.S., Çolak A., Aktaş Z., Dinçel A.S. (2019). Neuroprotective effects of topical coenzyme Q10 + vitamin E in mechanic optic nerve injury model. Eur. J. Ophthalmol.

[24] Ko M.-L., Peng P.-H., Hsu S.-Y., Chen C.-F. (2010). Dietary Deficiency of Vitamin E Aggravates Retinal Ganglion Cell Death in Experimental Glaucoma of Rats. Curr. Eye Res..

[25] Cellini M., Caramazza N., Mangiafico P., Possati G.L., Caramazza R. (1998). Fatty acid use in glaucomatous optic neuropathy treatment. Acta Ophthalmol. Scand. Suppl..

[26] Costagliola C., Libondi T., Menzione M., Rinaldi E., Auricchio G. (1985). Vitamin E and red blood cell glutathione. Metabolism.

[27] Bartollino S., Palazzo M., Semeraro F., Parolini B., Caruso C., Merolla F., Guerra G., Costagliola C. (2020). Effects of an antioxidant protective topical formulation on retinal tissue of UV-exposed rabbits. Int. Ophthalmol..

[28] Sun Y., Erdem E., Lyu A., Zangalli C., Wizov S.S., Lo D., E Spaeth E., Richman J., Spaeth G.L. (2016). The SPARCS: A novel assessment of contrast sensitivity and its reliability in patients with corrected refractive error. Br. J. Ophthalmol..

[29] Gupta L., Cvintal V., Delvadia R., Sun Y., Erdem E., Zangalli C., Lu L., Wizov S.S., Richman J., Spaeth E. (2017). SPARCS and Pelli–Robson contrast sensitivity testing in normal controls and patients with cataract. Eye.

[30] Richman J., Spaeth G.L., Wirostko B. (2013). Contrast sensitivity basics and a critique of currently available tests. J. Cataract. Refract. Surg..

[31] Richman J., Zangalli C., Lu L., Wizov S.S., Spaeth E., Spaeth G.L. (2015). The Spaeth/Richman contrast sensitivity test (SPARCS): Design, reproducibility and ability to identify patients with glaucoma. Br. J. Ophthalmol..

[32] Nelson P., Aspinall P., Papasouliotis O., Worton B., O’Brien C. (2003). Quality of Life in Glaucoma and Its Relationship with Visual Function. J. Glaucoma.

[33] Rossi G.C.M. (2018). Age and Gender Influence Reaction to Glaucoma Diagnosis. Eur. Ophthalmic Rev..

[34] Floriani I., Quaranta L., Rulli E., Katsanos A., Varano L., Frezzotti P., Rossi G.C.M., Carmassi L., Rolle T., Ratiglia R. (2016). Health-related quality of life in patients with primary open-angle glaucoma. An Italian multicentre observational study. Acta Ophthalmol..

[35] Ang G.S., Fenwick E.K., Constantinou M., Gan A.T.L., Man R.E.K., Casson R.J., A Finkelstein E., Goldberg I., Healey P.R., Pesudovs K. (2020). Selective laser trabeculoplasty versus topical medication as initial glaucoma treatment: The glaucoma initial treatment study randomised clinical trial. Br. J. Ophthalmol..

[36] King A.J., Fernie G., Azuara-Blanco A., Burr J.M., Garway-Heath T., Sparrow J.M., Vale L., Hudson J., MacLennan G., McDonald A.M. (2018). Treatment of Advanced Glaucoma Study: A multicentre randomised controlled trial comparing primary medical treatment with primary trabeculectomy for people with newly diagnosed advanced glaucoma—study protocol. Br. J. Ophthalmol..

[37] Stewart W.C., Stewart J.A., Nelson L.A. (2011). Ocular Surface Disease in Patients with Ocular Hypertension and Glaucoma. Curr. Eye Res..

[38] Rossi G.C.M., Pasinetti G.M., Scudeller L., Bianchi P.E. (2013). Ocular Surface Disease and Glaucoma: How to Evaluate Impact on Quality of Life. J. Ocul. Pharmacol. Ther..

[39] Ekici F., Loh R., Waisbourd M., Sun Y., Martinez P., Nayak N., Wizov S.S., Hegarty S., Hark L.A., Spaeth G.L. (2015). Relationships Between Measures of the Ability to Perform Vision-Related Activities, Vision-Related Quality of Life, and Clinical Findings in Patients With Glaucoma. JAMA Ophthalmol..

[40] Fresina M., Dickmann A., Salerni A., De Gregorio F., Campos E.C. (2007). Effect of oral CDP-choline on visual function in young amblyopic patients. Graefe’s Arch. Clin. Exp. Ophthalmol..

[41] Rejdak R., Toczołowski J., Solski J., Duma D., Grieb P. (2002). Citicoline Treatment Increases Retinal Dopamine Content in Rabbits. Ophthalmic Res..

[42] Spalletta G., Cravello L., Gianni W., Piras F., Eiorio M., Ecacciari C., Casini A.R., Echiapponi C., Sancesario G., Fratangeli C. (2016). Homotaurine Effects on Hippocampal Volume Loss and Episodic Memory in Amnestic Mild Cognitive Impairment. J. Alzheimer’s Dis..

[43] Davinelli S., Chiosi F., Di Marco R., Costagliola C., Scapagnini G. (2017). Cytoprotective Effects of Citicoline and Homotaurine against Glutamate and High Glucose Neurotoxicity in Primary Cultured Retinal Cells. Oxidative Med. Cell. Longev..

[44] Goyal A., Srivastava A., Sihota R., Kaur J. (2014). Evaluation of Oxidative Stress Markers in Aqueous Humor of Primary Open Angle Glaucoma and Primary Angle Closure Glaucoma Patients. Curr. Eye Res..

