# Supplementary material for: Effects of Citicoline, Homotaurine, and Vitamin E on Contrast Sensitivity and Visual-Related Quality of Life in Patients with Primary Open-Angle Glaucoma: A Preliminary Study
Source: Molecules. 2020 Nov 29;25(23):5614. doi: 10.3390/molecules25235614 (PMC7730471; doi:10.3390/molecules25235614)
Supplement: Supplementary file 1 [file molecules-25-05614-s001.pdf]

The Glaucoma Quality of Life-15 questionnaire

**Does your vision give you any difficulty, even with glasses, with the following activities?**	**None**	**A little bit**	**Some**	**Quite a lot**	**Severe**	**Do not perform for nonvisual reasons**
Reading newspapers	1	2	3	4	5	0
Walking after dark	1	2	3	4	5	0
Seeing at night	1	2	3	4	5	0
Walking on uneven ground	1	2	3	4	5	0
Adjusting to bright lights	1	2	3	4	5	0
Adjusting to dim lights	1	2	3	4	5	0
Going from light to dark room or vice versa	1	2	3	4	5	0
Tripping over objects	1	2	3	4	5	0
Seeing objects coming from the side	1	2	3	4	5	0
Crossing the road	1	2	3	4	5	0
Walking on steps/stairs	1	2	3	4	5	0
Bumping into objects	1	2	3	4	5	0
Judging distance of foot to step/curb	1	2	3	4	5	0
Finding dropped objects	1	2	3	4	5	0
Recognizing faces	1	2	3	4	5	0
